# Responses of a locust visual interneuron correlate with simple and compound object motion within the vertical plane

**DOI:** 10.1242/jeb.250488

**Published:** 2025-10-22

**Authors:** Zandra M. Santa Rita, Sinan Zhang, John R. Gray

**Affiliations:** ^1^ Department of Biology, University of Saskatchewan, 112 Science Place, Saskatoon, SK, Canada, S7N 5E2; ^2^ Department of Curriculum and Pedagogy, University of British Columbia, 2125 Main Mall, Vancouver, BC, Canada, V6T 1Z4; ^3^ Department of Medical Genetics, Cumming School of Medicine, University of Calgary, Calgary, AB, Canada, T2N 4N1

**Keywords:** Collision detection, DCMD, Locust, Vertical motion, Vision

## Abstract

Animals living in complex visual environments must contend with multiple visual cues that signal potential threats by detecting approaching objects and generating adaptive avoidance responses. One collision detection system in locusts is composed of the lobula giant movement detector (LGMD) and its postsynaptic partner, the descending contralateral movement detector (DCMD). Extensive work on this pathway has revealed that it is preferentially selective to visual stimuli generated by an approaching object (looming) and triggers avoidance behaviours such as jumping and flight steering. Recent work has shown that this pathway also responds characteristically to complex object motion that includes trajectory changes in the horizontal plane. To test the hypothesis that responses of this pathway correlate to motion in the vertical plane, we recorded from the DCMD while presenting combinations of simple looming as well as translation and transitions from and to looming. We found that the DCMD responses occurred earlier and were more robust for vertical translation in the lateral visual field, perpendicular to the centre of the eye. We also found strong correlations for the timing and firing rate as well as the duration and number of spikes of described phases of the response. These findings fit with our existing understanding of how this pathway conveys visual information to downstream elements initiating and controlling escape behaviours and provide further information on how this tractable system adds to our understanding of fundamental mechanisms that underly visually evoked behaviours.

## Teaser

**Summary:** Activity of a locust visual neural pathway correlates with the azimuthal position of simple and compound object motion in the vertical plane.

## INTRODUCTION

Animals living in complex visual environments require robust detection systems to detect and respond to threats such as colliding objects and attacking predators. Behaviours and underlying neural correlates for dealing with these threats have been studied across a wide range of taxa including: primates (Maier et al., 2004), mice (De Franceschi et al., 2016), gerbils (Ellard, 2004), birds (Sun and Frost, 1998; Cao et al., 2004), frogs (Yamamoto et al., 2003), fish (Gallagher and Northmore, 2006; Preuss et al., 2006; Dunn et al., 2016), crabs (Medan et al., 2015; Carbone et al., 2018) and insects (Robertson and Johnson, 1993; Jabłoński and Strausfeld, 2001; Verspui and Gray, 2009; Yamawaki and Toh, 2009b). Visual motion can occur across 360 deg of azimuth and elevation of the visual field, and animals such as insects have visual systems that can detect motion across a large range of angles (Krapp and Gabbiani, 2005). Moreover, objects can move in one or more planes, including horizontal and vertical planes. This requires that visual detection systems possess sensitivity to motion within single or multiple planes or trajectory changes across planes.

The locust is a well-studied tractable system for investigating underlying neural mechanisms of visually guided escape behaviours. Locusts reliably respond to approaching objects with stereotypical jumping (Santer et al., 2005; Fotowat et al., 2011; Parkinson et al., 2020) or flight steering manoeuvres (Santer et al., 2012). These behaviours have been investigated at the level of the underlying muscles that produce them (Fotowat and Gabbiani, 2007; Chan and Gabbiani, 2013; McMillan et al., 2013) as well as neurons that detect visual stimuli generated by approaching objects. One of the best studied visual pathways in locusts that responds to visual properties of approaching objects is the lobula giant movement detector (LGMD) and its postsynaptic partner, the descending contralateral movement detector (DCMD). The LGMD receives retinotopic inputs from the compound eyes (O'Shea and Rowell, 1976) and encodes object expansion through spike rate modulation (Schlotterer, 1977) that is conveyed to the DCMD in a 1-to-1 spike ratio (Rind, 1984). The DCMD descends along the contralateral side of the body and synapses onto interneurons and motor neurons that control the legs and wings (Pearson et al., 1985; Simmons, 1980). As such, the LGMD/DCMD pathway is strongly implicated in initiating and controlling visually evoked avoidance behaviours (Fotowat et al., 2011; Santer et al., 2012) and has been the subject of extensive modelling (Rind and Bramwell, 1996; Gabbiani et al., 2002; Wang et al., 2018; Zhu et al., 2018; Olson et al., 2021).

While the LGMD/DCMD preferentially responds to looming objects approaching along a direct collision course (Rind and Simmons, 1992), it also responds to translation motion across the visual field (Rogers et al., 2010; McMillan and Gray, 2012) and its firing rate is modulated by stimulus dynamics from objects that change trajectory in the horizontal plane. This modulation is represented by a transient firing rate decrease as an object transitions from translation to looming or a transient firing rate increase as an object transitions from looming to translation (McMillan and Gray, 2012). Additionally, these modulations are influenced by the location of the trajectory and the transition in the visual field. Although the DCMD responds to motion in the vertical plane (Rogers et al., 2010), it is not known whether trajectory changes in the vertical plane evoke similar firing rate modulation that occurs as a result of changes in the horizontal plane. Visual motion presented to locusts on the ground would be primarily in the dorsal visual field and vertical motion could be an important cue for initiating a jump. Flying locusts would receive visual motion information from dorsal and ventral regions of the visual field, which would be important in evoking flight steering. To further characterize DCMD responses to behaviourally relevant visual motion stimuli, we presented locusts with object motion along trajectories in the vertical plane. These included combinations of looming, translation and transitions from or to looming. These stimuli tested the hypothesis that DCMD firing rate parameters correlate with the azimuthal portion of vertical motion. We found that DCMD firing indeed correlated with azimuthal position, such that motion at a more lateral azimuthal angle, perpendicular to the centre of the eye, tended to evoke earlier and more robust responses. These findings are discussed in the context of a growing knowledge base of this system and the implications for the initiation of avoidance behaviour.

## MATERIALS AND METHODS

### Animals

We used 20 adult male *Locusta migratoria* (Linnaeus 1758) reared in a crowded colony maintained in the Department of Biology at the University of Saskatchewan. Locusts were raised at 25–28°C on a 12 h:12 h light:dark cycle with experiments performed during the locusts' light cycle to avoid any modifications in neural responses that could arise from night cycle activity. Experiments were carried out at room temperature (approximately 25°C).

### Preparation

The locust's legs were removed, and the wings were restrained preceding the attachment of a rigid tether to the ventral thorax using low melting point beeswax. A segment of ventral cervical cuticle was removed to expose a portion of the ventral nerve cord anterior to the prothoracic ganglion. Following nerve cord exposure, the locust was moved to the recording stage. We used a single silver wire hook electrode, insulated with a mixture of Vaseline and mineral oil, to record activity from the left ventral nerve cord, contralateral to the right eye. A silver wire ground electrode was inserted into the abdomen. Locusts were then positioned dorsal side up, with the rostral end 12 cm away from and facing the apex of a rear projection dome screen (diameter 72 cm). We designated the coordinate system such that 0 deg was directly in front of the locust, +90 deg was at the centre of the right eye and −90 deg was at the centre of the left eye. Locusts were left unstimulated against a solid white background for at least 5 min before visual stimulation.

### Stimulus generation

We used similar computer-generated visual objects to those described previously (Stott et al., 2018). Specifically, we created image sequences of a 14 cm virtual black sphere (luminance 1 cd m^−2^) travelling at 3 m s^−1^, which can be described by the ratio *l*/|*v*|=23 ms, where *l* is the half size of the sphere and |*v*| is the absolute velocity (*v*) (Gabbiani et al., 1999). We presented the sphere against a white background (luminance 172 cd m^−2^) using the Pyglet library in the Python programming language and projected images onto the dome screen using an InFocus DepthQ projector (with colour wheel removed) and a GeForce GTX 660 video card. Images were rendered at 100 frames s^−1^ and the code accounted for dome curvature to render simulated objects with the correct perspective. For all stimuli, the final image remained on the screen for 1 s. Within the Pyglet code, we rendered each stimulus frame such that the calculated subtense angle (θ) of the sphere was:
(1)

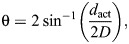
where *d*_act_ is the actual diameter of the sphere (14 cm) and *D* is the distance of the centre of the sphere to the locust eye. Because the locust was positioned 12 cm from the apex of the dome (radius 31 cm), we were able to create a final subtense angle of 180 deg, thus accurately producing a perceived time of collision.

**Fig. 1.**
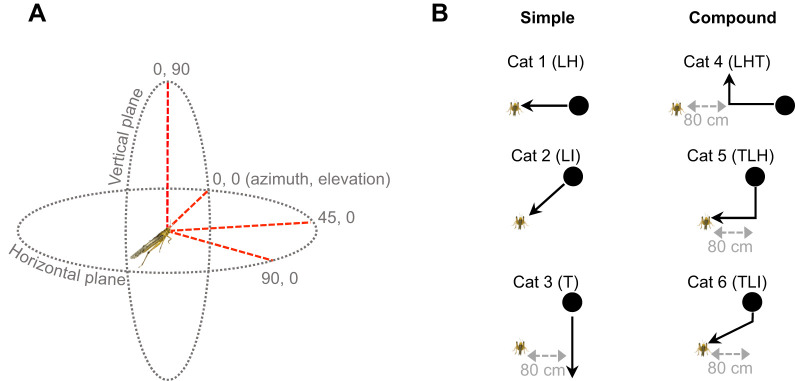
**Coordinate system and visual stimulus categories.** (A) Coordinate system based on azimuthal and elevation positions such that 0 deg azimuth and 0 deg elevation (0,0) was directly in front of the head of the locust. 0 deg elevation represents the horizontal plane and 0 deg azimuth represents the vertical plane (see Materials and Methods for details). For example, (90,0) represents an azimuthal angle 90 deg relative to the locust's right eye at an elevation of 0 deg (horizontal plane). For clarity, the coordinate system is shifted slightly to the right and above the locust. (B) General stimulus categories of simple (left) or compound (right) trajectories at 90 deg azimuth. Locusts are shown from behind. LH, looming within the horizontal plane; LI, looming within a plane inclined at 45 deg elevation; T, translation starting above the horizontal plane; LHT, initial looming within the horizontal plane transitioning to translation; TLH, initial translation (from above in this example), transitioning to looming within the horizontal plane; TLI, translation (from above in this example), transitioning to looming within an inclined plane. Details of motion at different azimuthal angles and in reciprocal directions are shown in Fig. S1.

We designated the three-dimensional coordinate system for object motion based on azimuthal and elevation angles where 0 deg azimuth and 0 deg elevation (0,0) was directly in front of the head of the locust (Fig. 1A). We designated 0 deg of elevation as the horizontal plane and 0 deg of azimuth as the vertical plane. In the horizontal plane, azimuthal angles were to the right (0, 45 or 90 deg) of the front of the locust's head and in the vertical plane, elevation angles were either above (positive) or below (negative) the horizontal plane. Downward motion (D) began at a positive elevation angle whereas upward motion (U) began at a negative elevation angle. We created six general stimulus categories based on the direction of motion (Fig. 1B). Stimuli travelled along simple (one trajectory) or compound (two trajectories) paths of motion. Generally, for simple trajectories, category 1 (cat 1) consisted of looming motion within the horizontal plane (looming horizontal, LH). Category 2 (cat 2) consisted of looming motion within a plane inclined (loom inclined, LI) at 45 or −45 deg elevation, above or below the horizontal plane, respectively. Category 3 (cat 3) consisted of translation motion (T) that passed through the horizontal plane at 0 deg elevation at a distance of 80 cm from the locust's eye in either a downward (TD) or upward (TU) direction. For compound trajectories, category 4 (cat 4) consisted of initial looming motion within the horizontal plane that then transitioned to translation motion at a distance of 80 cm from the locust's eye to either positive or negative elevation (loom horizontal to translation, LHT). Category 5 (cat 5) consisted of initial translational motion from positive or negative elevation that then transitioned at 80 cm from the locust's eye to looming within the horizontal plane (translation to loom horizontal, TLH). Category 6 (cat 6) consisted of initial translational motion from positive or negative elevation that transitioned at 45 or −45 deg elevation to looming within an inclined plane (translation to loom inclined, TLI). For each category, we presented stimuli at 0, 45 or 90 deg azimuth. We designated acronyms for each stimulus based on the initial direction of motion, the azimuthal angle and the final direction of motion (Fig. S1). Examples for each category include: LH45 – looming within the horizontal plane at 45 deg azimuth (cat 1), LID45 – looming from positive elevation within an inclined plane in a downward direction at 45 deg azimuth (cat 2), TD45 – translation from a positive elevation in a downward direction at 45 deg azimuth (cat 3), LH45TU – looming within the horizontal plane at 45 deg azimuth and then transitioning to translation in an upward direction (cat 4), TD45LH – translation from positive elevation in a downward direction at 45 deg azimuth and then transitioning to looming in the horizontal plane (cat 5), and TD45LID – translation from positive elevation in a downward direction at 45 deg azimuth and then transitioning to looming in an inclined plane in a downward direction (cat 6). For categories 2–6, we also presented stimuli in reciprocal directions. Thus, we presented 33 unique stimuli (Fig. S1) and we replicated each presentation three times to each locust (*n*=20). Each stimulus and replicate were presented in a random order unique for each locust and we used a 3 min inter-stimulus interval to avoid neural habituation.

The initial dataset included stimuli from categories 2–6. Therefore, each locust was presented with a total of three replicates of 30 unique stimuli for a total of 90 stimuli. Following data collection, we identified a miscalculation of the stimulus timing for cat 6 stimuli at 45 deg azimuth. Specifically, the virtual distance of transition to looming was not consistent with comparable stimuli at 0 deg and 90 deg azimuth. Therefore, we collected data from a second group of 20 locusts that included the correct transition distances as well as stimuli in cat 1. Each locust was presented with three replicates of five unique stimuli as well as a single direct loom at 90 deg azimuth at the start and end of each stimulus sequence to test for electrode stability throughout the recording session. Therefore, for the second dataset, each locust was presented with 17 stimuli. Including both datasets, Table S1 and Fig. S1 describe detailed parameters for 33 unique motion stimuli that varied within the horizontal and vertical planes at 0, 45 or 90 deg azimuth.

Individual pixel sizes on the projection screen were approximately 0.49 mm^2^, corresponding to a visual subtense angle (θ) of ∼0.4 deg, which is below the 1 deg resolution of individual ommatidia (Horridge, 1978). For all stimuli, we identified the time that the object passed through a subtense angle of 1 deg and designated this as the time that the stimulus started (*t*_s_), based on the locust's ability to detect the object. For stimuli in categories 1, 2, 5 and 6 (stimuli that ended with a looming trajectory), a 1 ms square wave synchronization pulse produced by the Python code identified the time of collision (*t*_c_). For stimuli in cat 3 (simple translational motion), the synchronization pulse identified the time that the object passed through the horizontal plane at 0 deg elevation (*t*_0_), and for stimuli in cat 4 (compound trajectories that ended with translation), the synchronization pulse identified the time of transition (*t*_t_). We also identified *t*_t_ for categories 5 and 6, which were 260 ms and 400 ms before *t*_c_, respectively.

### Neural recordings

**Fig. 2.**
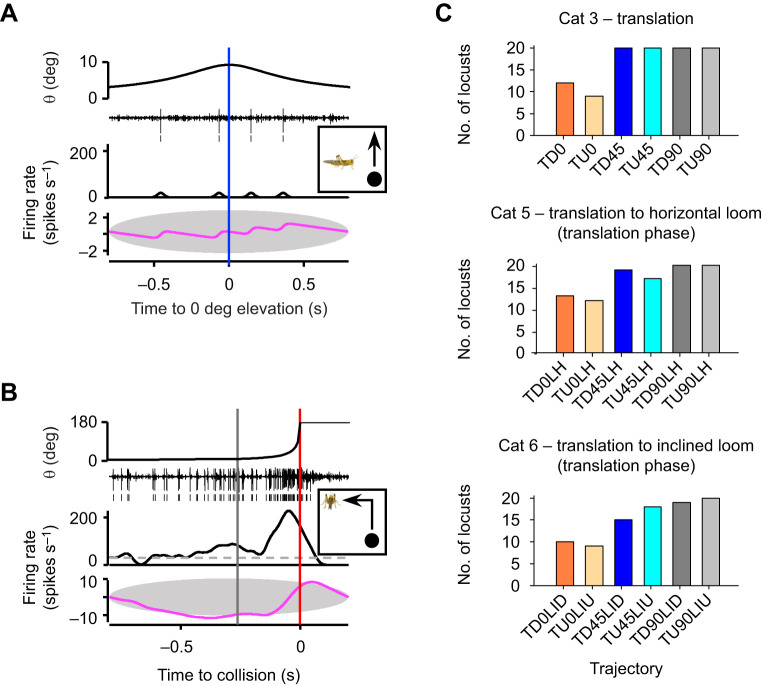
**Descending contralateral movement detector (DCMD) responses to different object trajectories.** Sample responses of the same locust to translation from below at 0 deg azimuth (A) and translation from below transitioning to looming in the horizontal plane at 90 deg azimuth (B). Plots, from top to bottom in A and B, show the object subtense angle (θ), the raw DCMD recording, rasters of DCMD spike times, peristimulus time histogram (PSTH; 95% confidence interval (CI) of the histogram, dashed line shown in B), and cumulative sum of the histogram (pink line) against a 99% CI ellipse (grey shade). Insets show the object trajectory. (C) The number of locusts responding to each trajectory for translation motion in category (cat) 3 (top) and the translation phase of motion for compound trajectories in cat 5 (middle) and cat 6 (bottom). For definitions, see Table S1.

We recorded neural activity from the left ventral nerve cord (Fig. 2) for each stimulus and amplified the signal with a differential AC amplifier (A-M Systems, model no. 1700, 100 Hz high pass and 5 kHz low pass filters, gain 100×). We sampled recordings at 20 kHz, digitized with a Data Translation DT9818 data acquisition board (TechmaTron Instruments, Inc., Laval, QC, Canada) interfaced with DataView version 11 acquisition and analysis software (W. J. Heitler, University of St Andrews), and used threshold detection in DataView to discriminate DCMD spikes, which are the largest within the ventral nerve cord.

### Spike train analysis

**Fig. 3.**
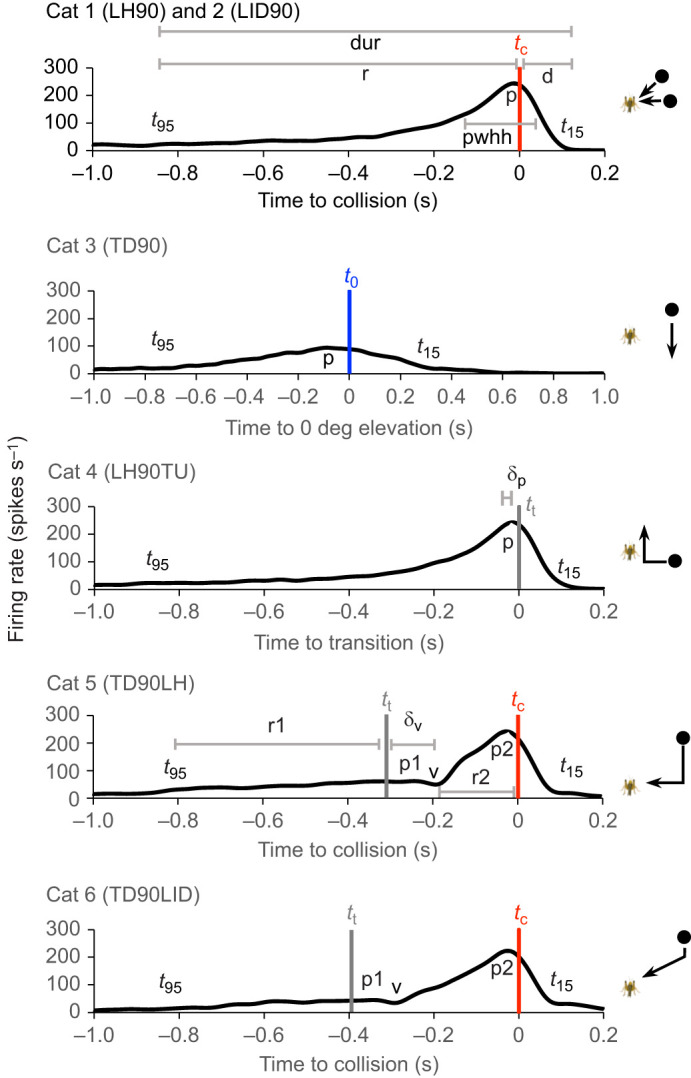
**Firing parameters of the DCMD in response to different object motion.** Sample mean PSTHs from 20 locusts for each stimulus category. Red, blue and dark grey vertical lines indicate time of collision (*t*_c_), time the object passed through 0 deg (*t*_0_) elevation and time of transition (*t*_t_), respectively. Grey horizontal lines with end caps indicate the duration of the respective parameter. Diagrams to the right indicate the respective object trajectory relative to the locust. See Table S2 for parameter details.

We created peristimulus time histograms (PSTHs) from DCMD spike times using Neuroexplorer spike train analysis software (NEX Technologies, Plexon Inc., Dallas, TX, USA) using a 1 ms bin width and a 50 ms Gaussian smoothing filter (Guest and Gray, 2006). To determine whether the DCMD responded to a stimulus (Fig. 2), we determined whether the PSTH crossed the 95% confidence interval (CI) of the histogram and whether the cumulative sum exceeded the boundaries of a 99% CI ellipse (Ritzmann et al., 2008). For trials in which the DCMD responded (Fig. 3; Table S2) we first calculated the time, relative to *t*_c_, at which the histogram last increased above the 95% CI with a positive slope (*t*_95_) and the time, relative to *t*_c_ when the firing decreased to 15% of the maximum value (*t*_15_). All of the specific firing parameters measured across all stimuli included: (1) timing (*t*) – the time, relative to *t*_c_ (cat 1, 2, 5, 6), *t*_0_ (cat 3) or *t*_t_ (cat 4), of the firing rate at the single (*t*_p_) or second (*t*_p2_) peak, the time of the first peak (*t*_p1_), the time of the local minima (valley, *t*_v_) associated with a trajectory change (cat 5 and 6), and *t*_15_; (2) firing rate (*f*) – the firing rate at the single (*f*_p_) or second (*f*_p2_) peak associated with *t*_c_, *t*_0_ or *t*_t_), the firing rate at the first peak (*f*_p1_), the firing rate at the valley (*f*_v_), and the normalized firing rate change (n*f*_v_) from *t*_t_ to *t*_v_; (3) durations – the total response duration from *t*_95_ to *t*_15_, the peak width at half the maximum fire rate at each peak, the rise phase (r) for each peak, the decay phase (d) from time of peak (*t*_p_ or *t*_p2_) to *t*_15_, and the response delay (δ) from the time of transition to either a peak (cat 4) or valley (cat 5 and 6); and (4) number of spikes – for the entire response (*t*_95_−*t*_15_), from *t*_95_ to firing at each peak, from peak firing to *t*_15_, from a transition associated peak firing to *t*_v_, and from *t*_v_ to the next peak firing. Some firing parameters could not be measured from all locusts presented with motion in all azimuthal planes, thus affecting the sample sizes in subsequent analyses. The sample sizes for each parameter, represented as the number of locusts, are reported in Table 1.

**Table 1.** Firing parameter sample sizes

Parameter	Cat 1	Cat 2		Cat 3		Cat 4		Cat 5		Cat 6	
		D	U	D	U	D	U	D	U	D	U
Time											
*t*_p/p2_	20	20	20	12,20,20	9,20,20	20	20	20	20	20	20
*t*_p1_								10,20,20	12,20,20	10,14,20	9,18,20
*t*_15_	20	20	20	12,20,20	9,20,20	20	20	20	20	20	20
*t*_v_								13,19,20	12,19,20	10,14,20	3,18,20
Firing rate											
*f*_p/p2_	20	20	20	12,20,20	9,20,20	20	20	20	20	20	20
*f*_p1_								13,19,20	12,19,20	10,14,20	9,18,20
*f*_t_						20	20	20	20	20	20
*f*_v_								13,19,20	12,19,20	10,14,20	3,18,20
*f*′ (n*f*_v_)								13,19,20	12,19,20	10,14,20	3,18,20
Duration											
dur	20	20	20	12,20,20	9,20,20	20	20	20	20	20	20
pwhh/pwhh_p2_	20	20	20	12,20,20	9,20,20	20	20	20	20	20	20
pwhh_p1_								13,19,20	12,19,20	10,14,20	9,18,20
r	20	20	20	12,20,20	9,20,20	20	20				
r1								13,19,20	12,19,20	9,14,20	9,18,20
r2								13,19,20	12,19,20	9,14,20	3,18,20
d	20	20	20	12,20,20	9,20,20	20	20	20	20	20	20
d1								13,19,20	12,19,20	11,14,20	3,18,20
δ_p_						20	20				
δ_v_								13,19,20	12,19,20	10,14,20	9,18,20
No. of spikes											
sp*_t_*__95_–*t*_15__	20	20	20	12,20,20	9,20,20	20	20	20	20	20	20
sp*_t_*__95_–*t*_p_/*t*_p2__	20	20	20	12,20,20	9,20,20	20	20	20	20	20	20
sp*_t_*__p/p2_–*t*_15__	20	20	20	12,20,20	9,20,20	20	20	20	20	20	20
sp*_t_*__95_–*t*_p1__								13,19,20	12,19,20	9,14,20	9,18,20
sp*_t_*__p1_–*t*_v__								13,19,20	12,19,20	9,14,20	3,18,20
sp*_t_*__v_–*t*_p2__								13,19,20	12,19,20	9,14,20	3,18,20

Summary of sample sizes of measured descending contralateral movement detector (DCMD) firing parameters (for definitions, see Table S2). The stimulus category (cat; for definitions, see Table S1) is indicated at the top, with the direction of motion for the translation phase of the stimulus below (D, down; U, up). Triple values indicate the number of locusts from which the parameter could be measured for motion at 0, 45 and 90 deg azimuth, respectively; otherwise, parameters were measured for all 20 locusts.

### Statistical analysis

We tested data for normality and equal variance with SigmaPlot 14.5 (Systat Software, Richmond, CA, USA). We used a chi-squared test to determine whether the number of locusts responding to a stimulus was affected by the trajectory type and/or azimuth position of the sphere. Data measured from DCMD firing properties did not satisfy assumptions of normality or equal variance, and therefore we used a Spearman correlation analysis to assess relationships between the azimuthal angle and stimulus trajectory with DCMD response properties. Table 2 shows the coefficient values for parameters measured. For some trajectories (e.g. TU0), object motion did not evoke any response from the DCMD. Therefore, the dataset consisted of unequal sample sizes. Significant correlations were assessed at *P*<0.05. Conventions for the correlation coefficient (ρ) considered 0 to ±0.09 to be non-correlative and ±0.1 to ±0.29, ±0.3 to ±0.49 and ±0.5 to ±1 to be small, medium and large correlations, respectively (Cohen, 2013).

**Table 2.** Spearman correlation coefficients

Parameter	Cat 1	Cat 2		Cat 3		Cat 4		Cat 5		Cat 6	
		D	U	D	U	D	U	D	U	D	U
Time (Figs 5 and 6)											
*t*_p/p2_	−0.64	−0.65	−0.86	n.s.	n.s.	−0.87	−0.90	−0.84	−0.92	−0.53	−0.81
*t*_p1_								n.s.	n.s.	n.s.	−0.56
*t*_15_	n.s.	n.s.	n.s.	0.66	0.48	n.s.	−0.49	−0.43	−0.49	n.s.	n.s.
*t*_v_								n.s.	−0.51	n.s.	−0.59
Firing rate (Fig. 7)											
*f*_p/p2_	0.40	0.48	0.52	0.89	0.69	0.71	0.75	0.63	0.62	0.36	0.65
*f*_p1_								0.88	0.62	0.72	0.61
*f*_t_						0.74	0.75	0.88	0.80	0.84	0.79
*f*_v_								0.82	0.85	0.70	0.78
*f*′ (n*f*_v_)								0.59	0.52	0.50	0.81
Duration (Fig. 8)											
dur	0.76	0.81	0.79	0.79	0.72	0.89	0.82	0.54	0.50	0.62	0.72
pwhh/pwhh_p2_	0.61	0.63	0.84	0.56	0.55	0.88	0.85	0.69	0.72	0.51	0.74
pwhh_p1_								0.73	n.s.	0.39	0.35
r	0.67	0.84	0.77	0.35	0.55	0.86	0.83				
r1								0.39	0.43	n.s.	0.35
r2								−0.70	−0.37	n.s.	n.s.
d	0.43	0.46	0.49	0.65	0.54	0.76	n.s.	n.s.	n.s.	0.42	0.38
d1								n.s.	n.s.	n.s.	0.40
δ_p_						−0.87	−0.90				
δ_v_								n.s.	−0.51	n.s.	−0.59
No. of spikes (Fig. 9)											
sp*_t_*__95_–*t*_15__	0.87	0.88	0.86	0.90	0.84	0.93	0.89	0.86	0.85	0.84	0.88
sp*_t_*__95_–*t*_p_/*t*_p2__	0.81	0.90	0.88	0.83	0.67	0.93	0.91	0.88	0.86	0.82	0.88
sp*_t_*__p/p2_–*t*_15__	0.67	0.60	0.64	0.84	0.72	0.87	0.74	0.42	0.56	0.57	0.63
sp*_t_*__95_–*t*_p1__								0.78	0.78	0.57	0.69
sp*_t_*__p1_–*t*_v__								0.66	0.38	0.33	0.64
sp*_t_*__v_–*t*_p2__								0.72	0.74	0.60	0.80

Summary of Spearman correlation coefficients from linear regressions between the stimulus azimuthal angle and measured DCMD firing parameters (for definitions, see Table S2). The formatting is the same as for Table 1: the stimulus category (cat; for definitions, see Table S1) is indicated at the top, with the direction of motion for the translation phase of the stimulus below (D, down; U, up). n.s. indicates that the correlation was not significant. Data are plotted in Figs 5–9.

## RESULTS

### Motion in the frontal visual field evokes fewer responses

For all locusts, the DCMD responded to looming along a simple trajectory or the looming phase of a compound trajectory. However, depending on the azimuthal position, not all locust DCMDs responded to simple translation motion or the translation phases of compound trajectories. Fig. 2A shows a trial in which the DCMD did not respond to upward translation motion from below at 0 deg azimuth (cat 3 – TU0), as indicated by the cumulative sum of the PSTH remaining within the 99% CI ellipse. However, in the same locust, the DCMD responded robustly to translation motion from below at 90 deg azimuth that transitioned to looming in the horizontal plane (cat 5 – TU90LH; Fig. 2B). Chi-squared analysis showed that the number of locusts responding to simple translation motion or the translation phase of compound trajectories with an initial looming component varied by trajectory (Fig. 2C). Specifically, significantly fewer locusts responded to simple translation in either direction (down or up) at 0 deg azimuth (cat 3 – χ^2^_5_=46.84, *P*<0.001) or the translation phase (down or up) of compound trajectories at 0 or 45 deg azimuth (cat 5 – χ^2^_5_=23.58, *P*<0.001, cat 6 – χ^2^_5_=30.24, *P*<0.001). These findings suggest that the DCMD is less sensitive to vertical translation motion in the frontal field of view.

### General trajectory-dependent DCMD response properties

**Fig. 4.**
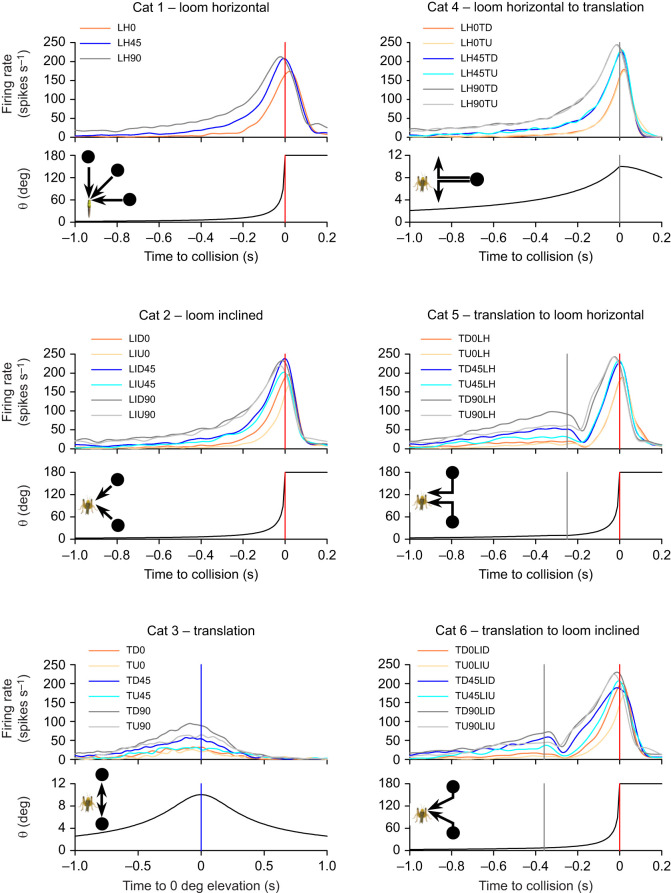
**Mean DCMD responses for all stimulus trajectories.** Specific trajectories (Table S1) are indicated in each panel key. Orange, blue and grey lines are responses to motion at 0, 45 and 90 deg, respectively. Darker shaded lines of the same colour are responses from appropriate trajectories in which motion begins above the horizontal plane and lighter shades are responses in which motion begins below the horizontal plane. For each stimulus category, the top plots are the PSTHs, the bottom plots show the object subtense angle (θ) and the diagram indicates an example of the trajectory. Trajectory details are described in Fig. S1. *n*=20 locusts except for cat 3 (TDO=12, TU0=9).

Mean PSTHs (Fig. 4) show that the stimulus category, azimuthal angle of motion and direction of motion relative to the vertical plane affected motion-evoked DCMD firing rates. For all categories, motion along increased (more lateral) azimuthal angles evoked earlier and more robust responses. Objects that travelled along simple trajectories (cat 1–3) or transitioned to a translating trajectory following looming (cat 4) evoked an increasing firing rate with a single peak near the closest position to the locust, which was *t*_c_ for cat 1 and 2, *t*_0_ for cat 3 and *t*_t_ for cat 4. For cat 5 and 6, a transition from translation to looming evoked a firing rate that peaked near transition (*t*_t_), decreased to a minimum (valley) associated with *t*_t_, which subsequently increased to a peak near collision (*t*_c_). Whereas the general PSTH shape with each category was similar across all azimuthal planes of motion, peaks and valleys represented higher firing rates that occurred earlier for more lateral azimuthal angles.

Motion above or below the horizontal plane evoked different responses depending on the category and azimuthal angle. For cat 2, looming from below along an inclined plane (LIU) evoked lower peak firing at 0 and 45 deg azimuth compared with approaches from above. There was no apparent effect of looming from above or below at 90 deg azimuth. For translating objects (cat 3), motion from below evoked lower peaks associated with *t*_0_ at 45 and 90 deg azimuth, whereas there was no apparent effect of motion direction at 0 deg azimuth. For objects that transitioned to a translation trajectory following looming (cat 4), there was no apparent effect of the azimuthal position or final direction of motion. For objects that transitioned to looming in the horizontal plane following vertical translation (cat 5), responses to the translation phase of motion at different azimuthal angles were consistent with those from cat 3, whereas responses to the looming phase appeared not to be affected by the initial direction of motion. For objects that transitioned to looming along an inclined plane following translation (cat 6), responses to the translation phase of motion at different azimuthal angles were consistent with those from cat 3 and 5, and responses to the looming phase were consistent with those from cat 2.

### Example of correlation analysis

**Fig. 5.**
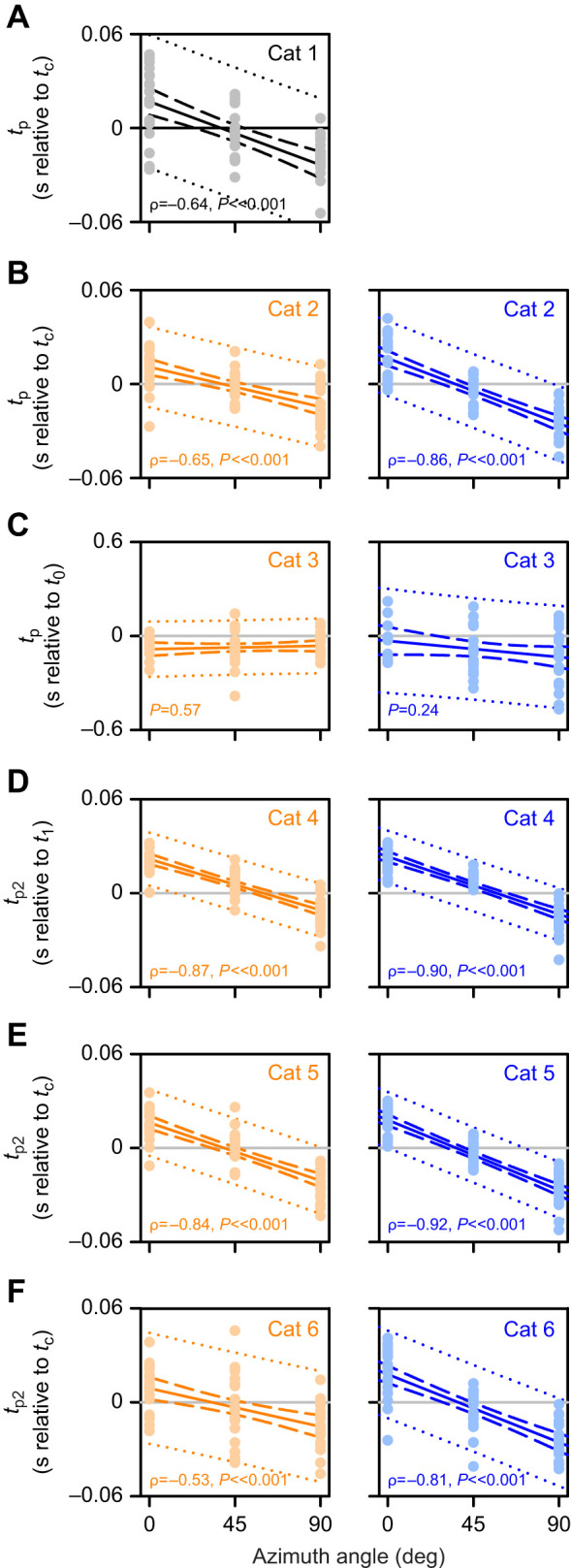
**Linear regressions and Spearman coefficients for the time of the peak (*t*_p_) or second peak (*t*_p_2__) of DCMD firing.** *t*_p_ (A–D) and *t*_p_2__ (E,F) data are from stimulus cat 1–6, respectively. For B–F, orange and blue symbols and lines are from stimuli with a downward or upward phase of motion, respectively. Light shaded symbols represent the mean values of the three trials from each locust, pooled across the entire dataset. Dark lines represent the linear regression (solid), the 95% CI (dashed) and the 95% prediction interval (dotted). The Spearman coefficient (ρ) and *P*-value are included for each plot of the pooled data.

To assess general correlations between the azimuthal angle of the stimulus trajectory and DCMD firing parameters, we calculated the mean value from three trials for each locust and then calculated the Spearman correlation coefficients using the parameter means pooled from all locusts. Because we were not able to measure firing parameters from all locusts in all azimuthal planes (Table 1), we were not able to calculate the correlation coefficient for each locust and therefore used pooled data to test across the entire dataset and report the linear regression, 95% CI and 95% predicted interval to establish the statistical validity of the regression. Example plots (Fig. 5) show data, regressions and correlation coefficients for the time of peak firing, relative to the time of collision, for the single (Fig. 5A–D, cat 1–4) or second (Fig. 5E,F, cat 5 and 6) peak. The data revealed significant strong negative correlations (ρ<−0.5) for cat 1, 2 and 4–6, demonstrating that the time of the peak occurred earlier in response to trajectories along more lateral azimuthal angles. The correlations were consistent for downward or upward phases of motion. For stimulus cat 3 (Fig. 5C, translation), we observed no significant correlation with azimuthal angle, irrespective of the direction of motion.

### Timing

**Fig. 6.**
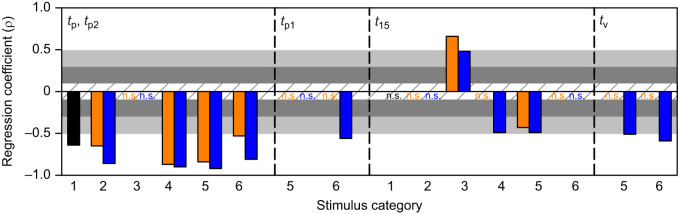
**Spearman correlation coefficients for DCMD timing parameters.** The specific DCMD response parameter is indicated top left. The leftmost panel shows coefficients for the time of the single peak (cat 1–4) or the second peak (cat 5 and 6) firing rates. Orange and blue bars and letters are from stimuli with a downward or upward phase of motion, respectively (cat 2–6). n.s., non-significant correlation. The cross-hatched, dark grey, light grey and white horizontal bands represent non-correlative, small, medium and large correlations, respectively (see Materials and Methods).

Fig. 6 shows correlation coefficients for all timing parameters measured. The leftmost subpanel (*t*_p_/*t*_p2_) is from the same coefficient values as in Fig. 5. The only significant result we observed for the timing of the first peak (*t*_p1_) in response to a compound trajectory (cat 4–6) was a strong negative correlation with the azimuthal trajectory from cat 6 (inclined looming following an upward translation). For simple looming stimuli, the end time of the response (*t*_15_) was not affected by the azimuthal trajectory of direct looms (cat 1–2), whereas for cat 3 (translation), *t*_15_ showed a strong positive correlation with downward translation and a medium positive correlation with upward translation. For compound trajectories, we observed a medium negative correlation for cat 4 with looming following an upward translation. For cat 5 and 6, *t*_15_ showed a medium negative correlation for horizontal looms following downward or upward transition (cat 5) but was not correlated with the azimuthal angle for inclined looms following either direction of translation (cat 6). The time of the valley of the firing rate (*t*_v_) showed strong negative correlations for a direct (cat 5) or inclined (cat 6) loom following upward translation. These data suggest that the timing of DCMD firing parameters was differentially affected by the azimuthal angle of the stimulus trajectory and the direction of the translation phase of motion.

### Firing rate

**Fig. 7.**
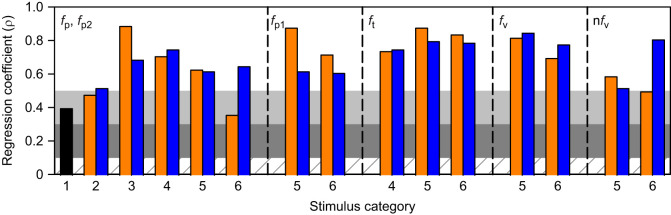
**Spearman correlation coefficients for DCMD firing rate parameters.** The specific DCMD response parameter is indicated top left. The leftmost panel shows coefficients for the maximum firing rate at the single peak (cat 1–4) or the second peak (cat 5 and 6). Orange and blue bars are from stimuli with a downward or upward phase of motion, respectively (cat 2–6). The cross-hatched, dark grey, light grey and white horizontal bands are as described in Fig. 6.

Fig. 7 shows that the firing rate parameters measured increased with the azimuthal angle of all stimulus trajectories. All correlations were strong except for medium correlations for the single peak (*f*_p_) in response to a direct horizontal loom (cat 1) or direct inclined loom from above (cat 2), and for the second peak (*f*_p2_) in response to an inclined loom following translation from above (cat 6).

### Duration

**Fig. 8.**
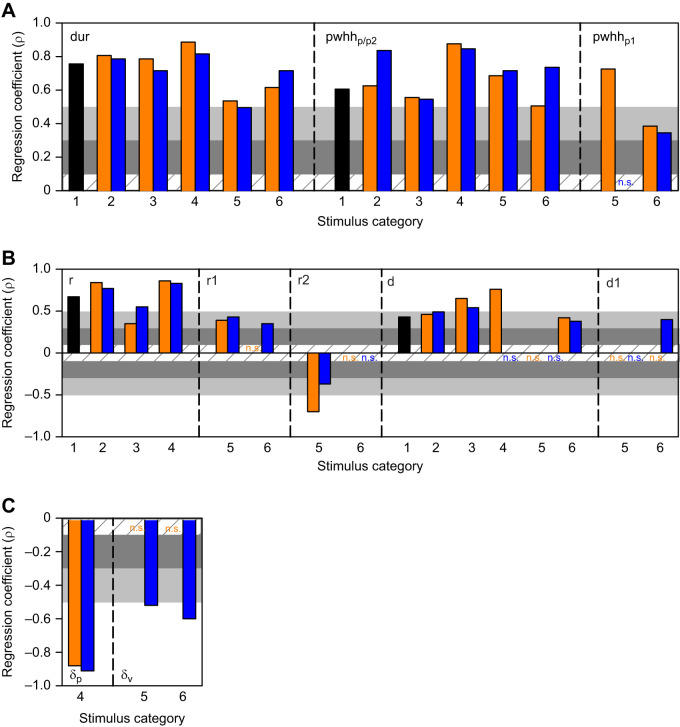
**Spearman correlation coefficients for DCMD duration parameters.** The specific DCMD response parameter is indicated top left. ( A) Coefficients for the total response duration and peak width at half height (pwhh) of the single peak (p – cat 1–4), second peak (p2 – cat 5 and 6), or first peak (p1 – cat 5 and 6). (B) Coefficients for the single rise phase (r – cat 1–4), first rise phase (r1 – cat 5 and 6) and second rise phase (r2 – cat 5 and 6). (C) Coefficients for the delay time from the time of transition to the time of the single peak (d_p_ – cat 4) or the time of transition to the valley (d_v_ – cat 5 and 6). Orange and blue bars and letters are from stimuli with a downward or upward phase of motion, respectively (cat 2–6). n.s., non-significant correlation. The cross-hatched, dark grey, light grey and white horizontal bands are as described in Fig. 6.

The total response duration (dur), the peak width at half height for the single or second peak (pwhh_p/p2_) and the peak width at half height for the first peak (pwhh_p1_) increased significantly with the azimuthal angle of the stimulus trajectory for all stimuli trajectories, except for pwhh_p1_ for an upward translation that preceded a horizontal loom (cat 5), which was not correlated (Fig. 8A). All correlations were strong except for medium correlations for pwhh_p1_ in response to an inclined loom following translation from either direction (cat 6). The response rise phase to the single peak (r) and the first peak (r1) positively correlated with the azimuthal angle of the stimulus trajectory, except for r1 in response to downward translation that preceded an inclined loom (cat 6), which were not correlated (Fig. 8B). Correlations were strong except for medium correlations for r in response to downward translation (cat 3), and for significant correlations for transitions to direct or inclined looming irrespective of translation direction (cat 5 and 6). The response rise phase for the second peak (r2) showed a strong or medium negative correlation with a direct loom following downward or upward translation, respectively (cat 5). We observed no significant correlation of r2 for inclined looms following translation in either direction (cat 6).

Significant correlations for the decay phase of the DCMD response from the single or second speak (d) or from the first peak (d1) were all positive (Fig. 8B). For d, there was no significant correlation for cat 4 with upward translation or for cat 5 with either translation direction. For d1, the only significant correlation was for an inclined loom following upward translation (cat 6). Correlations were strong for d in response to translation in either direction (cat 3) and for translation upward following a direct loom (cat 4). All other correlations were moderate.

The delay from the time of trajectory transition to the single peak (δ_p_ – cat 4) or valley (δ_v_ – cat 5 and 6) showed strong negative correlations except for δ_v_ responses to direct or inclined looms following downward translation, which were not correlated (Fig. 8C).

These findings show that the overall and detailed duration of DCMD firing increased with the azimuthal angle of simple trajectories. However, these parameters were differentially affected by the azimuthal position of compound trajectories.

### Number of spikes

**Fig. 9.**
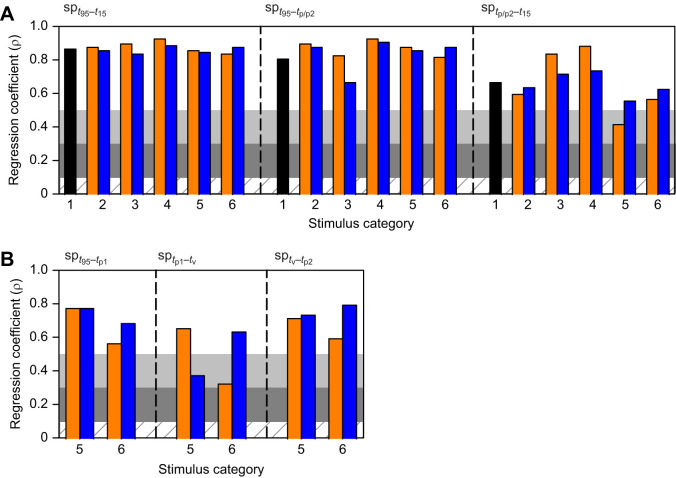
**Spearman correlation coefficients for DCMD duration parameters.** The specific DCMD response parameter is indicated top left. (A) Coefficients for the number of spikes for the total response duration (from *t*_95_ to *t*_15_), from the response start (*t*_95_) to the single peak (p – cat 1–4) or second peak (p2 – cat 5 and 6), and from the single or second peak to the time of the end of the response (*t*_15_). (B) Coefficients from stimulus cat 5 and 6 for the number of spikes from the start of the response (*t*_95_) to the first peak (*t*_p1_), *t*_p1_ to the time of the valley (*t*_v_), and *t*_v_ to the time of the second peak (*t*_p2_). Orange and blue bars are from stimuli with a downward or upward phase of motion, respectively (cat 2–6). The cross-hatched, dark grey, light grey and white horizontal bands are as described in Fig. 6.

The number of spikes for all measured stimulus intervals increased with the azimuthal angle of the stimulus trajectory (Fig. 9). All correlations were strong except for medium correlations of the interval from the time of the second peak (*t*_p2_) to *t*_15_ in response to horizontal looms following downward translation (cat 5; Fig. 9A) and the time of the first peak (*t*_p1_) to the time of the valley (*t*_v_) in response to a horizontal loom following upward translation (cat 5) or an inclined loom following downward translation (cat 6; Fig. 9B).

## DISCUSSION

We tested the hypothesis that DCMD firing properties correlate with trajectories of an object moving within a vertical plane. Specifically, we tested stimuli that moved along one of 33 unique trajectories that varied the azimuthal angle, direction of motion and the absence or presence of a trajectory change. For simple trajectories, we found that the DCMD responded to looming along a horizontal or inclined plane with a firing rate that increased during the approach and peaked near the time of collision. Simple translation evoked a firing rate increase to a peak that occurred before passing through the horizontal plane. Moreover, significantly fewer locusts responded to translation in the frontal visual field. For transitions from looming to translation, the firing rate increased to a peak near the time of transition that was consistent irrespective of the final translation direction. For transitions from translation to looming, the firing rate decreased to a local minimum (valley) and subsequently increased during the looming phase. We found that for all stimuli, responses were more robust for motion in the lateral visual field or in the upper visual field, especially for simple translation or the initial translation phase of a compound trajectory. Significant correlations of the stimulus azimuthal angle with the timing of important DCMD response stages were negative except for the end of the response (*t*_15_) to simple translation, which positively correlated with azimuth. Firing rates of specific DCMD response events and the number of spikes within defined response windows were all significantly and positively correlated with azimuthal angle. Significant correlations for the durations of described response windows positively correlated with azimuthal angle, except for the rising phase of the collision-related peak following horizontal translation, which was negatively correlated. Taken together, our results show that DCMD responses to object motion were affected by the azimuthal motion in the vertical plane and the direction of motion in either the upper or lower visual fields. Although we used two separate groups of locusts for the dataset (see Materials and Methods), the response profile of our presentation of a simple horizontal loom at 45 deg is comparable to that previously reported using the same stimulus (Stott et al., 2018). Therefore, though we needed to recalculate stimulus parameters for a subset of stimuli, the DCMD responses were accurately reflected in the entire dataset.

The results reported here are in general agreement with previous findings from locusts responding to motion primarily in the horizontal plane, suggesting common coding strategies irrespective of the plane of motion. Responses to simple looming trajectories within the horizontal plane reported here (Fig. 4, cat 1) are consistent with those from previous studies (Gabbiani et al., 1999; Gray et al., 2001; Matheson et al., 2004; Rind and Simmons, 1992; Stott et al., 2018). For simple looming along an inclined plane (Fig. 4, cat 2), we observed a higher and earlier peak firing rate in response to more lateral trajectories, and for trajectories at 0 and 45 deg azimuth, the DCMD was less sensitive to motion from below. Rogers et al. (2010) reported similar results using comparable stimuli. Fig. 4 (cat 3) revealed that for vertically translating trajectories, the azimuthal position and direction of motion affected DCMD responses, with the most robust responses evoked by motion from above at 90 deg azimuth. Though other studies have not investigated translation in the vertical plane, responses to horizontal translation were also directionally sensitive, with the strongest responses to motion starting in the anterior visual field (McMillan and Gray, 2012).

While this is the first study to examine locust DCMD responses to compound trajectories in the vertical plane, our results reflect responses to similar motion in the horizontal plane. Fig. 4 (cat 4) shows that deviation away from looming, with a trajectory change of 90 deg, evoked transition-related peak firing that occurred after transition for motion at 0 and 45 deg azimuth and before transition for motion at 90 deg azimuth. Deviations away from looming in the horizontal plane also evoked peaks that occurred either after transition, in which the trajectory change was <90 deg (McMillan and Gray, 2012; Yakubowski et al., 2016), or before transition, in which the trajectory changed by 90 deg (Yakubowski et al., 2016). Fig. 4 (cat 5 and 6) shows that deviation from translation to looming evoked a translation-related decrease in the firing rate (valley) that subsequently increased during the looming phase, irrespective of azimuthal position. Similar firing rate modulation was observed for deviation to looming in the horizontal plane (Dick and Gray, 2014; McMillan and Gray, 2012; Yakubowski et al., 2016).

The findings reported here are consistent with those from other studies on insects and other animals, suggesting common coding strategies for complex object motion in the visual field. In *Drosophila*, the giant fibre (GF) descending neuron receives velocity encoding inputs from lobula columnar type 4 (LC4) neurons and object size inputs from lobula plate/lobula columnar type 2 (LPLC2) neurons. Summing of these inputs onto GF activates a looming-triggered escape response (Ache et al., 2019). Other LC neurons encode distinct aspects of object motion, including looming, translation and complex motion, that integrate in downstream circuits involved in escape behaviours (Klapoetke et al., 2022). Foma-1 neurons, most sensitive to motion in the dorsal visual field, respond to a downward-moving small dot, and to looming, and are sufficient to elicit escape (de Vries and Clandinin, 2012). In the fly *Calliphora*, vertical translation cells that respond strongly to optic flow encode the fly's motion along near-cardinal axes that include horizontal and vertical motion (Longden et al., 2017). Additionally, vertical system cells that respond to large field motion also show local motion sensitivity responses to vertical motion (Krapp et al., 1998). In the mantis, looming-sensitive neurons showed similar responses to DCMD reported here and triggered defensive behaviour (Sato and Yamawaki, 2014). These neurons also responded to horizontal and vertical motion with firing rates that decreased when the object trajectory deviated from 0 deg azimuth (equivalent to our 90 deg azimuth) or 0 deg elevation (equivalent to our 0 deg elevation) (Yamawaki and Toh, 2009a). Additionally, the neurons were more responsive to upward motion than downward motion (Yamawaki and Toh, 2009b). Looming-sensitive neurons exist in the moth *Manduca sexta* (Wicklein and Strausfeld, 2000), and large field horizontal and vertical cells respond best to the respective motions of gratings (Wicklein and Varjú, 1999). Taken together, these findings demonstrate a rich complement of insect visual neurons that respond to complex visual motion across horizontal and vertical planes, and similar complements exist in other taxa. In crabs, the MLG1 neurons respond to looming (Oliva and Tomsic, 2014) and horizontal translation motion (Medan et al., 2007) and form a system of 16 retinotopically arranged elements that map 360 deg of azimuthal space (Medan et al., 2015). While neurons in the nucleus isthmi of fish respond preferentially to looming, they also respond, though more weakly, to horizontal translatory motion (Gallagher and Northmore, 2006). In the pigeon, looming-sensitive neurons in the nucleus rotundus (Sun and Frost, 1998), as well as large field neurons in the basal optic root, respond to vertical and horizontal translation (Wylie and Frost, 1999).

### Neural mechanisms of responses to complex vertical motion

A mechanistic explanation of our results that accounts for the optical density of the locust eye and the sensitivity of the LGMD across the visual field is consistent with previous reports for complex motion in the horizontal plane (McMillan and Gray, 2012; Dick and Gray, 2014). Translation motion in any plane results in a supralinear increase in the velocity of a leading edge across the ommatidia that leads to an increased firing rate (Fig. 4, cat 3). At the transition to looming, a subsequent decrease in leading edge expansion would reduce the spread of excitation through the network and allow feedforward inhibition to dominate, resulting in a transition-associated decrease in firing (valley; Fig. 4, cat 5 and 6). Excitation would then increase and dominate as looming continues, resulting in a loom-associated increase in the firing rate. A transition away from looming (Fig. 4, cat 4) would result in increased leading-edge expansion and a resulting increase in the firing rate at the time of transition that would quickly decrease as the object moves away from the locust.

Reduced responses to local motion in more frontal regions of the visual field (Krapp and Gabbiani, 2005) are consistent with our findings that the peak firing rate evoked by simple vertical translation positively correlates with more lateral azimuthal angles (Fig. 7, *f*_p_, cat 3) and reflects differences in optical density and DCMD sensitivity in these regions. However, the timing of the peaks was invariant across azimuthal angles. Firing rate differences are also reflected in the positive correlation of the valley firing rates (Fig. 7, *f*_v_). The optical density of the eye is greatest in the equatorial region of the frontal visual field whereas local motion sensitivity is greatest in the equatorial region of the caudolateral visual field and, in this region, correlates with the dorso-ventral optic density (Krapp and Gabbiani, 2005). Though differences in local motion sensitivity may compensate for optical density, the decreased sensitivity in the frontal visual field would explain the decreased responses that we found at 0 deg azimuth. Moreover, fewer locusts responded to vertical translation at 0 deg azimuth, suggesting a decrease or lack of responses directed in front of the locust. This is not surprising given that the DCMD is preferentially selective to looming and, therefore, less sensitive to translation (Rind and Simmons, 1992).

### Significance and future research

While we show that the LGMD/DCMD pathway responds characteristically to complex object motion and correlates with vertical motion at different azimuthal angles, future experiments would further define the neuronal mechanisms and behavioural consequences for these trajectories. For behaviour experiments, it would be valuable and possible to estimate a processing delay from stimulus input on the eye to the generation of behaviour. Gabbiani et al. (1999) report a delay of 15–35 ms from stimulus input to DCMD firing in the connective anterior to the suboesophageal connective and Gray et al. (2001) discuss a time of 214 ms from stimulus input to production of flight avoidance behaviour, which accounts for stimulus input, sensorimotor integration, neuromuscular drive and generation of wing kinematic changes. Because we recorded from the connective anterior to the prothoracic ganglia, we do not have an *a priori* estimate of a putative additional processing delay as spikes pass through the suboesophageal ganglion. Regardless, our intent was not to provide a detailed temporal analysis of the DCMD responses to the stimuli presented here. Rather, it was to test for correlations between DCMD activity and the azimuthal position of vertical motion.

It would be informative to record from both right and left DCMDs to determine whether motion in the frontal visual field is accounted for by the responses to both neurons simultaneously (Gabbiani et al., 2001). However, given that there is a significant, though reduced, response to translation at 0 deg azimuth for one DCMD, it is reasonable to assume the same for the other DCMD. Thus, translation motion directly in front of the locust may occur within a region of reduced sensitivity. It would also be important to record DCMD responses to trajectories presented here while in the presence of background visual flow, which affects responses to looming, translation and associated transitions in the horizontal plane (Silva et al., 2015; Yakubowski et al., 2016). To provide a behavioural context for vertical motion, it is possible to record escape responses such as jumping (Fotowat and Gabbiani, 2007; Fotowat et al., 2011) and flight (Santer et al., 2012; Chan and Gabbiani, 2013; McMillan et al., 2013) when presented with the trajectories used here. Motion in the dorsal visual field would be relevant for evoking jumping of locusts on the ground, whereas motion in the dorsal and ventral fields would be relevant for flight avoidance manoeuvres. Experiments to test these two working hypotheses could incorporate combinations of DCMD recordings, EMG recordings from leg or wing muscles, and kinematic analyses of leg or wing movements. These experiments would uncover underlying mechanisms of these behaviours, which is beyond the scope of the work reported here. The data presented here could also inform developing computational models of LGMD/DCMD responses to motion (Jones and Gabbiani, 2012; Olson et al., 2021) and lead to more robust algorithms that could be used in autonomous artificial systems.

## Supplementary Material

10.1242/jexbio.250488_sup1 Supplementary information

## References

[JEB250488C1] Ache, J. M., Polsky, J., Alghailani, S., Parekh, R., Breads, P., Peek, M. Y., Bock, D. D., Von Reyn, C. R. and Card, G. M. (2019). Neural basis for looming size and velocity encoding in the drosophila giant fiber escape pathway. *Curr. Biol.* 29, 1073-1081.e4. 10.1016/j.cub.2019.01.079 30827912

[JEB250488C2] Cao, P., Gu, Y. and Wang, S.-R. (2004). Visual neurons in the pigeon brain encode the acceleration of stimulus motion. *J. Neurosci.* 24, 7690-7698. 10.1523/JNEUROSCI.2384-04.2004 15342736 PMC6729630

[JEB250488C3] Carbone, J., Yabo, A. and Oliva, D. (2018). Characterization and modelling of looming-sensitive neurons in the crab Neohelice. *J. Comp. Physiol.* 204, 487-503. 10.1007/s00359-018-1257-1 29574596

[JEB250488C4] Chan, R. W. M. and Gabbiani, F. (2013). Collision-avoidance behaviors of minimally restrained flying locusts to looming stimuli. *J. Exp. Biol.* 216, 641-655. 10.1242/jeb.077453 23364572 PMC3561775

[JEB250488C5] Cohen, J. (2013). *Statistical Power Analysis for the Behavioral Sciences*, 2nd edn. New York: Routledge.

[JEB250488C6] De Franceschi, G., Vivattanasarn, T., Saleem, A. B. and Solomon, S. G. (2016). Vision guides selection of freeze or flight defense strategies in mice. *Curr. Biol.* 26, 2150-2154. 10.1016/j.cub.2016.06.006 27498569

[JEB250488C7] de Vries, S. E. J. and Clandinin, T. R. (2012). Loom-sensitive neurons link computation to action in the *Drosophila* visual system. *Curr. Biol.* 22, 353-362. 10.1016/j.cub.2012.01.007 22305754 PMC3298569

[JEB250488C8] Dick, P. C. and Gray, J. R. (2014). Spatiotemporal stimulus properties modulate responses to trajectory changes in a locust looming-sensitive pathway. *J. Neurophysiol.* 111, 1736-1745. 10.1152/jn.00499.2013 24478154

[JEB250488C69] Dunn, T. W., Gebhardt, C., Naumann, E. A., Riegler, C., Ahrens, M. B., Engert, F. and Bene, F. del (2016). Neural circuits underlying visually evoked escapes in larval zebrafish. *Neuron* 89, 613-628. 10.1016/j.neuron.2015.12.021 26804997 PMC4742414

[JEB250488C9] Ellard, C. G. (2004). Visually guided locomotion in the gerbil: a comparison of open- and closed-loop control. *Behav. Brain Res.* 149, 41-48. 10.1016/S0166-4328(03)00210-9 14739008

[JEB250488C10] Fotowat, H. and Gabbiani, F. (2007). Relationship between the phases of sensory and motor activity during a looming-evoked multistage escape behavior. *J. Neurosci.* 27, 10047-10059. 10.1523/JNEUROSCI.1515-07.2007 17855619 PMC2081158

[JEB250488C11] Fotowat, H., Harrison, R. R. and Gabbiani, F. (2011). Multiplexing of motor information in the discharge of a collision detecting neuron during escape behaviors. *Neuron* 69, 147-158. 10.1016/j.neuron.2010.12.007 21220105 PMC3035170

[JEB250488C12] Gabbiani, F., Krapp, H. G. and Laurent, G. (1999). Computation of object approach by a wide-field, motion-sensitive neuron. *J. Neurosci.* 19, 1122-1141. 10.1523/JNEUROSCI.19-03-01122.1999 9920674 PMC6782150

[JEB250488C13] Gabbiani, F., Mo, C. and Laurent, G. (2001). Invariance of angular threshold computation in a wide-field looming-sensitive neuron. *J. Neurosci.* 21, 314-329. 10.1523/JNEUROSCI.21-01-00314.2001 11150349 PMC6762430

[JEB250488C14] Gabbiani, F., Krapp, H. G., Koch, C. and Laurent, G. (2002). Multiplicative computation in a visual neuron sensitive to looming. *Nature* 420, 320-324. 10.1038/nature01190 12447440

[JEB250488C15] Gallagher, S. P. and Northmore, D. P. M. (2006). Responses of the teleostean nucleus isthmi to looming objects and other moving stimuli. *Visual Neurosci.* 23, 209-219. 10.1017/S0952523806232061 16638173

[JEB250488C16] Gray, J. R., Lee, J. K. and Robertson, R. M. (2001). Activity of descending contralateral movement detector neurons and collision avoidance behaviour in response to head-on visual stimuli in locusts. *J. Comp. Physiol. A* 187, 115-129. 10.1007/s003590100182 15524000

[JEB250488C17] Guest, B. B. and Gray, J. R. (2006). Responses of a looming-sensitive neuron to compound and paired object approaches. *J. Neurophysiol.* 95, 1428-1441. 10.1152/jn.01037.2005 16319198

[JEB250488C18] Horridge, G. A. (1978). The separation of visual axes in apposition compound eyes. *Philos. Trans. R. Soc. Lond. B Biol. Sci.* 285, 1-59. 10.1098/rstb.1978.0093 34179

[JEB250488C19] Jabłoński, P. G. and Strausfeld, N. J. (2001). Exploitation of an ancient escape circuit by an avian predator: Relationships between taxon-specific prey escape circuits and the sensitivity to visual cues from the predator. *Brain Behav. Evol.* 58, 218-240. 10.1159/000057565 11964498

[JEB250488C60] Jones, P. W. and Gabbiani, F. (2012). Logarithmic compression of sensory signals within the dendritic tree of a collision-sensitive neuron. *J. Neurosci.* 32, 4923-4934. 10.1523/jneurosci.5777-11.2012 22492048 PMC3752046

[JEB250488C20] Klapoetke, N. C., Nern, A., Rogers, E. M., Rubin, G. M., Reiser, M. B. and Card, G. M. (2022). A functionally ordered visual feature map in the Drosophila brain. *Neuron* 110, 1700-1711.e6. 10.1016/j.neuron.2022.02.013 35290791

[JEB250488C21] Krapp, H. G. and Gabbiani, F. (2005). Spatial distribution of inputs and local receptive field properties of a wide-field, looming sensitive neuron. *J. Neurophysiol.* 93, 2240-2253. 10.1152/jn.00965.2004 15548622

[JEB250488C22] Krapp, H. G., Hengstenberg, B. and Hengstenberg, R. (1998). Dendritic structure and receptive-field organization of optic flow processing interneurons in the fly. *J. Neurophysiol.* 79, 1902-1917. 10.1152/jn.1998.79.4.1902 9535957

[JEB250488C23] Longden, K. D., Wicklein, M., Hardcastle, B. J., Huston, S. J. and Krapp, H. G. (2017). Spike burst coding of translatory optic flow and depth from motion in the fly visual system. *Curr. Biol.* 27, 3225-3236.e3. 10.1016/j.cub.2017.09.044 29056452

[JEB250488C24] Maier, J. X., Neuhoff, J. G., Logothetis, N. K. and Ghazanfar, A. A. (2004). Multisensory Integration of Looming Signals by Rhesus Monkeys. *Neuron* 43, 177-181. 10.1016/j.neuron.2004.06.027 15260954

[JEB250488C25] Matheson, T., Rogers, S. M. and Krapp, H. G. (2004). Plasticity in the visual system is correlated with a change in lifestyle of *solitarious* and *gregarious* locusts. *J. Neurophysiol.* 91, 1-12. 10.1152/jn.00795.2003 13679397

[JEB250488C26] McMillan, G. A. and Gray, J. R. (2012). A looming-sensitive pathway responds to changes in the trajectory of object motion. *J. Neurophysiol.* 108, 1052-1068. 10.1152/jn.00847.2011 22572940

[JEB250488C27] McMillan, G. A., Loessin, V. and Gray, J. R. (2013). Bilateral flight muscle activity predicts wing kinematics and 3-dimensional body orientation of locusts responding to looming objects. *J. Exp. Biol.* 216, 3369-3380. 10.1242/jeb.087775 23737560

[JEB250488C28] Medan, V., Oliva, D. and Tomsic, D. (2007). Characterization of lobula giant neurons responsive to visual stimuli that elicit escape behaviors in the crab *Chasmagnathus*. *J. Neurophysiol.* 98, 2414-2428. 10.1152/jn.00803.2007 17715192

[JEB250488C29] Medan, V., Berón De Astrada, M., Scarano, F. and Tomsic, D. (2015). A network of visual motion-sensitive neurons for computing object position in an arthropod. *J. Neurosci.* 35, 6654-6666. 10.1523/JNEUROSCI.4667-14.2015 25926445 PMC6605188

[JEB250488C30] Oliva, D. and Tomsic, D. (2014). Computation of object approach by a system of visual motion-sensitive neurons in the crab Neohelice. *J. Neurophysiol.* 112, 1477-1490. 10.1152/jn.00921.2013 24899670

[JEB250488C31] Olson, E. G. N., Wiens, T. K. and Gray, J. R. (2021). A model of feedforward, global, and lateral inhibition in the locust visual system predicts responses to looming stimuli. *Biol. Cybern.* 115, 245-265. 10.1007/s00422-021-00876-8 33997912

[JEB250488C32] O'Shea, M. and Rowell, C. H. F. (1976). The neuronal basis of a sensory analyser, the acridid movement detector system. II. response decrement, convergence, and the nature of the excitatory afferents to the fan-like dendrites of the LGMD. *J. Exp. Biol.* 65, 289-308. 10.1242/jeb.65.2.289 187712

[JEB250488C33] Parkinson, R. H., Zhang, S. and Gray, J. R. (2020). Neonicotinoid and sulfoximine pesticides differentially impair insect escape behavior and motion detection. *Proc. Natl. Acad. Sci. USA* 117, 5510-5515. 10.1073/pnas.1916432117 32094166 PMC7071913

[JEB250488C34] Pearson, K. G., Boyan, G. S., Bastiani, M. J. and Goodman, C. S. (1985). Heterogeneous properties of segmentally homologous interneurons in the ventral nerve cord of locusts. *J. Comp. Neurol.* 233, 133-145. 10.1002/cne.902330108 3980770

[JEB250488C35] Preuss, T., Osei-Bonsu, P. E., Weiss, S. A., Wang, C. and Faber, D. S. (2006). Neural representation of object approach in a decision-making motor circuit. *J. Neurosci.* 26, 3454-3464. 10.1523/JNEUROSCI.5259-05.2006 16571752 PMC6673849

[JEB250488C36] Rind, F. C. (1984). A chemical synapse between two motion detecting neurones in the locust brain. *J. Exp. Biol.* 110, 143-167. 10.1242/jeb.110.1.143 6086803

[JEB250488C37] Rind, F. C. and Bramwell, D. I. (1996). Neural network based on the input organization of an identified neuron signaling impending collision. *J. Neurophysiol.* 75, 967-985. 10.1152/jn.1996.75.3.967 8867110

[JEB250488C38] Rind, F. C. and Simmons, P. J. (1992). Orhtopteran DCMD neuron: a reevaluation of responses to moving objects. I. selective responses to approaching objects. *J. Neurophysiol.* 68, 1654-1666. 10.1152/jn.1992.68.5.1654 1479436

[JEB250488C39] Ritzmann, R. E., Ridgel, A. L. and Pollack, A. J. (2008). Multi-unit recording of antennal mechano-sensitive units in the central complex of the cockroach, *Blaberus discoidalis*. *J. Comp. Physiol.* 194, 341-360. 10.1007/s00359-007-0310-2 18180927

[JEB250488C40] Robertson, R. M. and Johnson, A. G. (1993). Retinal image size triggers obstacle avoidance in flying locusts. *Naturwissenschaften (Berlin)* 80, 176-178. 10.1007/BF01226378

[JEB250488C41] Rogers, S. M., Harston, G. W. J., Kilburn-Toppin, F., Matheson, T., Burrows, M., Gabbiani, F. and Krapp, H. G. (2010). Spatiotemporal receptive field properties of a looming-sensitive neuron in solitarious and gregarious phases of the desert locust. *J. Neurophysiol.* 103, 779-792. 10.1152/jn.00855.2009 19955292 PMC2822700

[JEB250488C42] Santer, R. D., Yamawaki, Y., Rind, F. C. and Simmons, P. J. (2005). Motor activity and trajectory control during escape jumping in the locust *Locusta migratoria*. *J. Comp. Physiol.* 191, 965-975. 10.1007/s00359-005-0023-3 16044332

[JEB250488C43] Santer, R. D., Rind, F. C. and Simmons, P. J. (2012). Predator versus prey: locust looming-detector neuron and behavioural responses to stimuli representing attacking bird predators. *PLoS ONE* 7, e50146. 10.1371/journal.pone.0050146 23209660 PMC3507823

[JEB250488C44] Sato, K. and Yamawaki, Y. (2014). Role of a looming-sensitive neuron in triggering the defense behavior of the praying mantis *Tenodera aridifolia*. *J. Neurophysiol.* 112, 671-682. 10.1152/jn.00049.2014 24848471

[JEB250488C45] Schlotterer, G. R. (1977). Response of the locust descending movement detector neuron to rapidly approaching and withdrawing visual stimuli. *Can. J. Zool.* 55, 1372-1376. 10.1139/z77-179

[JEB250488C46] Silva, A. C., McMillan, G. A., Santos, C. P. and Gray, J. R. (2015). Background complexity affects the response of a looming-sensitive neuron to object motion. *J. Neurophysiol.* 113, 218-231. 10.1152/jn.00478.2014 25274344

[JEB250488C47] Simmons, P. J. (1980). Connexions between a movement-detecting visual interneurone and flight motoneurones of a locust. *J. Exp. Biol.* 86, 87-97. 10.1242/jeb.86.1.87

[JEB250488C48] Stott, T. P., Olson, E. G. N., Parkinson, R. H. and Gray, J. R. (2018). Three-dimensional shape and velocity changes affect responses of a locust visual interneuron to approaching objects. *J. Exp. Biol.* 221, jeb.191320. 10.1242/jeb.191320 30341087

[JEB250488C49] Sun, H. and Frost, B. J. (1998). Computation of different optical variables of looming objects in pigeon nucleus rotundus neurons. *Nat. Neurosci.* 1, 296-303. 10.1038/1110 10195163

[JEB250488C50] Verspui, R. and Gray, J. R. (2009). Visual stimuli induced by self-motion and object-motion modify odour-guided flight of male moths (*Manduca sexta* L.). *J. Exp. Biol.* 212, 3272-3282. 10.1242/jeb.031591 19801432

[JEB250488C51] Wang, H., Dewell, R. B., Zhu, Y. and Gabbiani, F. (2018). Feedforward inhibition conveys time-varying stimulus information in a collision detection circuit. *Curr. Biol.* 28, 1509-1521.e3. 10.1016/j.cub.2018.04.007 29754904 PMC5964032

[JEB250488C52] Wicklein, M. and Strausfeld, N. J. (2000). Organization and significance of neurons that detect change of visual depth in the hawk moth *Manduca sexta*. *J. Comp. Neurol.* 424, 356-376. 10.1002/1096-9861(20000821)424:2<356::AID-CNE12>3.0.CO;2-T 10906708

[JEB250488C53] Wicklein, M. and Varjú, D. (1999). Visual system of the European hummingbird hawkmoth *Macroglossum stellatarum* (sphingidae, lepidoptera): Motion-sensitive interneurons of the lobula plate. *J. Comp. Neurol.* 408, 272-282. 10.1002/(SICI)1096-9861(19990531)408:2<272::AID-CNE8>3.0.CO;2-9 10333274

[JEB250488C54] Wylie, D. R. W. and Frost, B. J. (1999). Responses of neurons in the nucleus of the basal optic root to translational and rotational flowfields. *J. Neurophysiol.* 81, 267-276. 10.1152/jn.1999.81.1.267 9914287

[JEB250488C55] Yakubowski, J. M., McMillan, G. A. and Gray, J. R. (2016). Background visual motion affects responses of an insect motion-sensitive neuron to objects deviating from a collision course. *Physiol. Rep.* 4, e12801. 10.14814/phy2.12801 27207786 PMC4886169

[JEB250488C56] Yamamoto, K., Nakata, M. and Nakagawa, H. (2003). Input and output characteristics of collision avoidance behavior in the frog *Rana catesbeiana*. *Brain Behav. Evol.* 62, 201-211. 10.1159/000073272 14573994

[JEB250488C57] Yamawaki, Y. and Toh, Y. (2009a). A descending contralateral directionally selective movement detector in the praying mantis *Tenodera aridifolia*. *J. Comp. Physiol.* 195, 1131-1139. 10.1007/s00359-009-0485-9 19888580

[JEB250488C58] Yamawaki, Y. and Toh, Y. (2009b). Responses of descending neurons to looming stimuli in the praying mantis *Tenodera aridifolia*. *J. Comp. Physiol.* 195, 253-264. 10.1007/s00359-008-0403-6 19093123

[JEB250488C59] Zhu, Y., Dewell, R. B., Wang, H. and Gabbiani, F. (2018). Pre-synaptic muscarinic excitation enhances the discrimination of looming stimuli in a collision-detection neuron. *Cell Rep.* 23, 2365-2378. 10.1016/j.celrep.2018.04.079 29791848 PMC5997271

