# Supplementary material for: Responses of a locust visual interneuron correlate with simple and compound object motion within the vertical plane
Source: J Exp Biol. 2025 Oct 22;228(20):jeb250488. doi: 10.1242/jeb.250488 (PMC12582412; doi:10.1242/jeb.250488)
Supplement: Supplementary information [file jexbio-228-250488-s1.pdf]

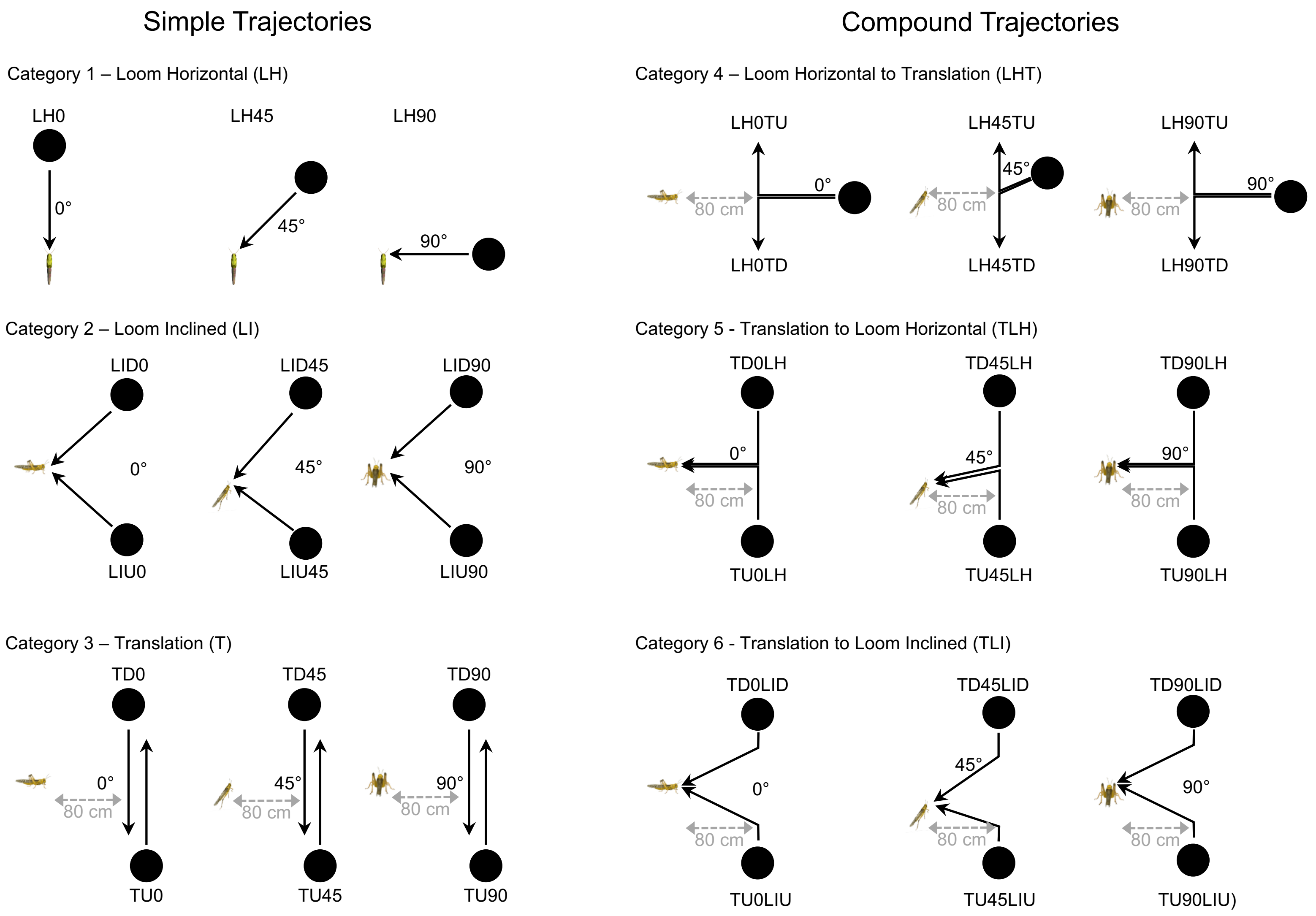
Simple Trajectories
Category 1 – Loom Horizontal (LH)
LH0
0°
LH45
45°
LH90
90°
Category 2 – Loom Inclined (LI)
LID0
0°
LIU0
LID45
45°
LIU45
LID90
90°
LIU90
Category 3 – Translation (T)
TD0
0°
80 cm
TU0
TD45
45°
80 cm
TU45
TD90
90°
80 cm
TU90
Compound Trajectories
Category 4 – Loom Horizontal to Translation (LHT)
LH0TU
0°
80 cm
LH0TD
LH45TU
45°
80 cm
LH45TD
LH90TU
90°
80 cm
LH90TD
Category 5 - Translation to Loom Horizontal (TLH)
TD0LH
0°
80 cm
TU0LH
TD45LH
45°
80 cm
TU45LH
TD90LH
90°
80 cm
TU90LH
Category 6 - Translation to Loom Inclined (TLI)
TD0LID
0°
80 cm
TU0LIU
TD45LID
45°
80 cm
TU45LIU
TD90LID
90°
80 cm
TU90LIU)

Fig. S1. Details of object motion. Object motion at 0°, 45°, or 90° azimuth for all stimulus categories. Category 1 shows the locust from above. Categories 2-6 show the locust from the right (0° azimuth), from slightly to the right and above (45° azimuth), or from behind (90° azimuth). For Categories 2-6, reciprocal directions are shown. In total, locusts were presented with 33 unique visual stimuli. LH = looming within the horizontal plane, LID = looming within an inclined plane from above horizontal in a downward direction, LIU = looming within an inclined plane from below horizontal in an upward direction, TD = translation from above horizontal in a downward direction, passing through the horizontal plane (elevation = 0°), TU = translation from below horizontal in an upward direction, passing through the horizontal plane. Acronyms for each stimulus represent the plane of object motion (Categories 1-3) or initial motion (Categories 4-6), the azimuthal angle of motion, and the second plane of object motion (Categories 4-6). For example, LHO (Category 1) represents looming in the horizontal plane at 0° azimuth and TD0LH (Category 5) represents initial translation downward at 0° azimuth, transitioning to looming within the horizontal plane.

Table S1. Stimulus parameters

Stimulus Designation	Initial trajectory	Final trajectory	Azimuthal angle
**Simple Trajectories**			
**Category 1 – Looming in Horizontal Plane (LH)**			
LH0	Loom Horizontal		0°
LH45	Loom Horizontal		45°
LH90	Loom Horizontal		90°
**Category 2 – Looming in Inclined Plane (LI)**			
LID0	Downward Loom		0°
LID45	Downward Loom		45°
LID90	Downward Loom		90°
LIU0	Upward Loom		0°
LIU45	Upward Loom		45°
LIU90	Upward Loom		90°
**Category 3 – Translation (T)**			
TD0	Downward Translation		0°
TD45	Downward Translation		45°
TD90	Downward Translation		90°
TU0	Upward Translation		0°
TU45	Upward Translation		45°
TU90	Upward Translation		90°
**Compound Trajectories**			
**Category 4 – Looming in Horizontal Plane to Translation (LH-T)**			
LH0TD	Loom horizontal	Downward Translation	0°
LH45TD	Loom horizontal	Downward Translation	45°
LH90TD	Loom horizontal	Downward Translation	90°
LH0TU	Loom horizontal	Upward Translation	0°
LH45TU	Loom horizontal	Upward Translation	45°
LH90TU	Loom horizontal	Upward Translation	90°
**Category 5 - Translation to Looming in Horizontal Plane (T-LH)**			
TD0LH	Downward Translation	Loom Horizontal	0°
TD45LH	Downward Translation	Loom Horizontal	45°
TD90LH	Downward Translation	Loom Horizontal	90°
TU0LH	Upward Translation	Loom Horizontal	0°
TU45LH	Upward Translation	Loom Horizontal	45°
TU90LH	Upward Translation	Loom Horizontal	90°
**Category 6 - Translation to Looming in Inclined Plane (T-LI)**			
TD0LID	Downward Translation	Downward Loom	0°
TD45LID	Downward Translation	Downward Loom	45°
TD90LID	Downward Translation	Downward Loom	90°
TU0LIU	Upward Translation	Upward Loom	0°
TU45LIU	Upward Translation	Upward Loom	45°
TU90LIU	Upward Translation	Upward Loom	90°

D = motion in downward direction, U = motion in upward direction

Details of the stimulus parameters demonstrated in Fig. S1. See Materials and Methods for general descriptions of stimulus categories.

Journal of Experimental Biology • Supplementary information

Table S2. DCMD firing parameters

Parameter	Description/Calculations	Category
**Time (t)**		
$t_{p/p2}$	Time of peak (p) or second peak (p_2) relative to t_c	1-4 or 5-6
t_{15}	Time crossing 15% of f_p or f_{p2} (response end)	1-4 or 5-6
t_{p1}	Time of first peak (p_1) relative to t_t	5-6
t_v	Time of valley (v) relative to t_t	5-6
**Firing rate (*f*)**		
$f_{p/p2}$	Firing rate at p or p_2	1-4 or 5-6
f_{p1}	Firing rate at p_1	5-6
f_t	Firing rate at transition	4-6
f_v	Firing rate at v	5-6
f'	Firing rate change from t_t to t_v (f_t - f_v/t_v - t_t)	5-6
**Durations**		
dur	Total response duration (t_{15} - t_{95})	1-6
pwhh/$pwhh_{p2}$	Peak width at half height for p or p_2	1-4 or 5-6
$pwhh_{p1}$	Peak width at half height for p_1	5-6
r	Rise phase to p (t_p - t_{95})	1-4
r_1	Rise phase to p_1 (t_{p1} - t_{95})	5-6
r_2	Rise phase to p_2 (t_{p2} - t_v)	5-6
d	Decay phase from t_p to t_{15} (t_{15} - t_p)	1-4
d_1	Decay phase from t_{p1} to t_v (t_v - t_{p1})	5-6
d_2	Decay phase from t_{p2} to t_{15} (t_{15} - t_{p2})	5-6
δ_p	Response delay from t_t to t_p (t_p - t_t) = t_p	4
δ_v	Response delay from t_t to t_v (t_v - t_t)	5-6
**Number of Spikes (sp)**		
$sp_{t95-t15}$	Number of spikes for entire response (t_{95} to t_{15})	1-6
$sp_{t95-tp/tp2}$	Number of spikes from t_{95} to t_p or t_{p2}	1-4 or 5-6
$sp_{tp/p2-t15}$	Number of spikes from t_p or t_{p2} to t_{15}	1-4 or 5-6
$sp_{t95-tp1}$	Number of spikes from t_{95} to t_{p1}	5-6
sp_{tp1-tv}	Number of spikes t_{p1} to t_v	5-6
sp_{tv-tp2}	Number of spikes t_v to t_{p2}	5-6

Detailed description of DCMD firing properties measured from peristimulus time histograms and the stimulus category for which they were measured.

Journal of Experimental Biology • Supplementary information
